# Harnessing CD16-Mediated NK Cell Functions to Enhance Therapeutic Efficacy of Tumor-Targeting mAbs

**DOI:** 10.3390/cancers13102500

**Published:** 2021-05-20

**Authors:** Cristina Capuano, Chiara Pighi, Simone Battella, Davide De Federicis, Ricciarda Galandrini, Gabriella Palmieri

**Affiliations:** 1 Department of Experimental Medicine, Sapienza University of Rome, 00161 Rome, Italy; cristina.capuano@uniroma1.it (C.C.); chiara.pighi@uniroma1.it (C.P.); simone.battella@reithera.com (S.B.); davide.defedericis@uniroma1.it (D.D.F.); 2 ReiThera Srl, 00128 Rome, Italy; 3 Department of Molecular Medicine, Sapienza University of Rome, 00161 Rome, Italy

**Keywords:** tumor-targeting mAb, memory NK cells, vaccinal effect, IFNγ, CD16, ADCC, combinatory immunotherapeutic approach

## Abstract

**Simple Summary:**

Natural Killer (NK) cells play a major role in cancer immunotherapy based on tumor-targeting mAbs. NK cell-mediated tumor cell killing and cytokine secretion are powerfully stimulated upon interaction with IgG-opsonized tumor cells, through the aggregation of FcγRIIIA/CD16 IgG receptor. Advances in basic and translational NK cell biology have led to the development of strategies that, by improving mAb-dependent antitumor responses, may overcome the current limitations of antibody therapy attributable to tolerance, immunosuppressive microenvironment, and genotypic factors. This review provides an overview of the immunotherapeutic strategies being pursued to improve the efficacy of mAb-induced NK antitumor activity. The exploitation of antibody combinations, antibody-based molecules, used alone or combined with adoptive NK cell therapy, will be uncovered. Within the landscape of NK cell heterogeneity, we stress the role of memory NK cells as promising effectors in the next generation of immunotherapy with the aim to obtain long-lasting tumor control.

**Abstract:**

Natural killer (NK) cells hold a pivotal role in tumor-targeting monoclonal antibody (mAb)-based activity due to the expression of CD16, the low-affinity receptor for IgG. Indeed, beyond exerting cytotoxic function, activated NK cells also produce an array of cytokines and chemokines, through which they interface with and potentiate adaptive immune responses. Thus, CD16-activated NK cells can concur to mAb-dependent “vaccinal effect”, i.e., the development of antigen-specific responses, which may be highly relevant in maintaining long-term protection of treated patients. On this basis, the review will focus on strategies aimed at potentiating NK cell-mediated antitumor functions in tumor-targeting mAb-based regimens, represented by (a) mAb manipulation strategies, aimed at augmenting recruitment and efficacy of NK cells, such as Fc-engineering, and the design of bi- or trispecific NK cell engagers and (b) the possible exploitation of memory NK cells, whose distinctive characteristics (enhanced responsiveness to CD16 engagement, longevity, and intrinsic resistance to the immunosuppressive microenvironment) may maximize therapeutic mAb antitumor efficacy.

## 1. Introduction

Therapeutic tumor-targeting monoclonal antibodies (mAbs) represent a very successful advancement in the treatment of several hematologic and solid tumors. Besides directly affecting tumor cell viability and/or proliferation, tumor-targeting mAbs activate several Fc-dependent effector mechanisms that importantly contribute to their therapeutic efficacy [1,2,3,4]. In this context, natural killer (NK) cells hold a critical position, due to the presence, on the vast majority of them, of CD16 (also named FcγRIIIA), the low-affinity receptor for IgG1 and IgG3. CD16 represents a prototype NK cell-activating receptor since its engagement is, by itself, sufficient to trigger cytotoxic activity and production of pro-inflammatory cytokines and chemokines, thus unleashing NK cell antitumor functions [4,5,6]. Human CD16, also expressed by macrophages and some circulating monocytes [7], consists of two extracellular Ig domains, a short cytoplasmic tail and a transmembrane domain that, in NK cells, allows its association with CD3ζ and FcεRIγ chains; these immunoreceptor tyrosine-based activation motif (ITAM)-containing subunits connect the receptor to intracellular signal transduction pathways, which coordinate the reorganization of the actin and microtubule cytoskeleton, and the activation of several transcription factors [5,8]. In NK cells, these events enable the antibody-dependent cell-mediated cytotoxicity (ADCC), which is accomplished by the targeted release of perforin- and granzyme-containing cytotoxic granules, and by engagement of Fas ligand and TNF-related apoptosis-inducing ligand (TRAIL) death receptors [9,10,11,12,13]. CD16 ligation also affects NK cell survival and proliferation [14,15] and induces the release of cytokines and chemokines, which promote the recruitment and activation of tumor-infiltrating immune cells [5,16,17,18]. In this regard, NK-derived interferon (IFN) γ stands as a well-recognized key immunoregulatory factor in the shaping of adaptive immune responses by promoting the activation of macrophages and dendritic cells (DC), as well as the generation of T helper (Th) 1 and cytotoxic T lymphocyte (CTL) responses [19,20,21,22]. In this regard, NK cell immunoregulatory role may contribute to the well-known capability of tumor-targeting mAbs to promote and amplify the development of long-lasting antitumor adaptive responses, a phenomenon that has been termed the “vaccinal effect” [17,23]. Finally, NK cells are exquisitely sensitive to therapeutic mAb-dependent activation since they only express an activating receptor for IgG (i.e. CD16) differently from all the other innate immunity effectors that also display inhibitory FcγR isoforms [24].

NK cells belong to the innate arm of antitumor immunosurveillance. Their capability to recognize transformed cells directly is a highly relevant but still only partially exploited characteristic from the perspective of improving the therapeutic efficacy of tumor-targeting mAb-based strategies. Indeed, NK cells are equipped with an array of germline-encoded activating receptors that cooperatively regulate their capability to discriminate between healthy and diseased cells [6,8,25,26,27,28,29,30]; NK cells’ ability to establish multiple activating interactions with mAb-opsonized tumor cells may amplify CD16-initiated functions, especially under conditions of suboptimal receptor aggregation. On the other hand, the direct or CD16-dependent antitumor effector functions of NK cells are restrained by the action of a wide array of MHC class I-specific and nonspecific inhibitory receptors that negatively regulate their interaction with tumor cells and/or other components of the tumor microenvironment (TME) [6,8,31,32,33,34,35,36].

Collectively, the exploiting of combinatory treatments targeting activating and/or inhibitory receptors to fine-tune tumor-specific mAb-induced NK cell activation is already actively investigated and will be further pursued in the future [37].

Finally, the phenotypic and functional heterogeneity of the circulating NK cell pool is increasingly appreciated. In particular, recent studies have revealed the capability of NK cells to adapt to environmental factors and to differentiate in long-lived specialized populations with enhanced effector functions, named “memory” or “adaptive”. This novel information may pave the way to better exploitation of NK cells to augment the therapeutic efficacy of mAb-based antitumor regimens, also in an adoptive cell therapy perspective [38,39].

In this review, we focus on several strategies aimed at potentiating NK cell-mediated antitumor functions in mAb-based therapeutic settings, which range from the modifications of therapeutic molecules or regimens (including mAb combinations, Fc-engineering, and the design of bi- or trispecific NK cell engagers (NKCE)) to the selection and genetic manipulation of NK effectors. In this context, we present the peculiar features that render memory NK cells an attractive tool to overcome the barriers to the broad application of NK cells for the ultimate benefit of cancer patients.

## 2. NK Cells as Participants to Antitumor Immunosurveillance

The involvement of NK cells in antitumor innate responses is well established, both in experimental models and in patients [16,17,21,25,26,27,28,40,41,42,43]. Indeed, NK cells are equipped with an array of activating receptors that recognize a multitude of still partially undefined ligands on tumor cells [6,8,16,44,45]. Among them, natural killer group 2D (NKG2D) and DNAX accessory molecule-1 (DNAM-1) recognize stress-induced cell surface molecules that are frequently overexpressed on neoplastic cells: NKG2D ligands belong to MIC (MHC I polypeptide-related sequences) and ULBP (UL16 binding protein) families, while CD155 (poliovirus receptor) and CD112 (Nectin 2), members of the nectin family, are recognized by DNAM-1. In addition, the C-type lectin CD94/NKG2C receptor complex and the members of the natural cytotoxicity receptor (NCR) family, i.e., NKp30, NKp44, and NKp46, induce activating signals in NK cells, upon binding to nonclassical HLA-E molecule or to still partially identified tumor-associated ligands, respectively [6,25,26,27,28,44,45,46,47,48,49]. Interestingly, NK cell-activating receptors also enable the recognition of cancer stem cells (CSCs), a self-renewing cell population that contributes to both tumor evolution and therapy resistance [50,51,52]. Further, the still poorly characterized capability of NK cells to react against senescent cells is an area of increasing interest in the context of antitumor immunosurveillance. Indeed, senescent cells produce a complex array of soluble factors, collectively named senescence-associated secretory phenotype (SASP), that profoundly modify tissue homeostasis and contribute to the establishment of a tumor-permissive inflammatory microenvironment. The reported capability of NK cells to recognize and eliminate the senescent tumor and, perhaps more importantly, stromal cells would importantly favor the shaping of an immunogenic tumor infiltrate [53,54].

On the other hand, NK cell activation and antitumor functions are restrained by inhibitory signals initiated by killer cell immunoglobulin-like receptor (KIRs) and CD94/NKG2A heterodimer upon recognition of classical and nonclassical MHC class I molecules, respectively [6]. In this regard, many studies have stressed the importance of mismatch at KIR/ligand interface in dictating NK cell-mediated protection against hematological malignancies in the setting of allogeneic hematopoietic stem cell transplantation (HSCT) [55]. Moreover, NK cells share the expression of several immune checkpoints with T cells, including programmed death-1 (PD-1), T-cell immunoglobulin and mucin domain containing-3 (TIM-3), and T-cell immunoreceptor with Ig and ITIM domains (TIGIT), which negatively regulate NK cell activation and functions upon interaction with ligands expressed on tumor cells and/or other components of the TME [8,31,32,33,34,35,36].

Due to the expression of several chemokine receptors, including CXCR3-CXCR4, CX_3_CR1, and CCR3-CCR5, circulating NK cells can be effectively recruited to tumor sites [56,57,58]. Increasing evidence suggests that NK presence within the tumor mass is a strong positive prognostic factor in several types of solid tumors, such as colorectal (CRC), renal, and head and neck squamous cell (HNSCC) carcinomas, and neuroblastoma [40,48,59,60,61,62,63,64]. Additionally, mature cytotoxic NK cells have been shown to be recruited to/retained selectively in metastatic lymphnodes of melanoma patients, and their responsiveness to activating cytokines could be exploited in novel immunotherapeutic strategies [65,66].

On the other hand, tumor-infiltrating NK cells may show an altered phenotype associated with profound functional defects, under the influence of an immunosuppressive TME. Several cell populations at the tumor site, such as myeloid-derived suppressor cells (MDSC), tumor-associated macrophages, tumor-associated fibroblasts, regulatory T cells (Treg), and tumor cells themselves, have been shown to negatively affect NK cell activation and effector functions in hepatocellular carcinoma, CRC, melanoma, etc. This immunosuppressive activity relies on the production of a plethora of cytokines such as IL-4, IL-10, or TGFβ, or metabolites such as ROS or prostaglandin E2; the establishment of a hypoxic microenvironment, or the depletion of trophic cytokines and nutrients, the latter occurring in an indoleamine 2,3-dioxygenase (IDO)-dependent manner, also contribute to dampening NK cell antitumor functions. All these factors may directly interfere with NK cell metabolism, activation, effector functions, or efficient infiltration of the tumor bed; additionally, they may indirectly hamper NK cell activation and functions by inducing the downregulation of activating receptors and/or promoting the upregulation of immune checkpoint ligands [25,26,40,43,46,64,67,68,69,70,71,72,73,74,75,76,77]. In particular, it was reported that the extent of NKp46, NKp30, and NKG2D downmodulation correlates with tumor progression in human cervical cancer, breast cancer, CRC, and non-small-cell lung carcinoma [67].

## 3. CD16 as a Relevant Receptor for Tumor-Targeting mAb Therapeutic Efficacy: The Contribution of NK Cells

Several studies have demonstrated that CD16-triggered ADCC and phagocytosis, mediated by NK cells and monocyte/macrophages, respectively, are the principal immune-dependent mechanisms through which tumor-targeting mAbs may mediate tumor cell killing and attain therapeutic efficacy [78].

Studies in FcγR-deficient mice showed that mAb-induced therapeutic effects require the FcεRIγ adaptor chain, which is involved in signal transduction of both FcγRI and FcγRIII expressed on monocytes, macrophages, and NK cells [1,2,3,4,17,24,79,80,81,82,83]. The role of CD16 is further supported by the observation that the clinical response to therapeutic mAb treatments is affected by a single nucleotide polymorphism (SNP) of the FCGR3A gene that affects binding affinity for IgG; indeed, patients that are homozygous for high-affinity allelic variant (FcγRIIIA-158-V/V) display better clinical outcome to therapeutic mAb regimens than those heterozygous (FcγRIIIA-158-V/F), or homozygous for low-affinity variant (FcγRIIIA-158-F/F), in follicular (FL) and non-Hodgkin’s lymphomas, CRC, and breast cancer [84,85,86,87,88]. This correlation has been observed in many but not all studies [89,90,91,92], suggesting that cohort characteristics, other patient’s genotypic factors, and/or treatment regimen can affect the impact of FcγR binding affinity on mAb therapeutic response; in this regard, copy number variability (CNV) of FCGR3A gene, influencing expression levels on the cell surface, may also contribute to interindividual variability in the response to mAb treatment [4,93,94].

Several lines of evidence support a crucial role for NK cells in the CD16-dependent therapeutic efficacy of tumor-targeting mAbs. The composition and quality of tumor-infiltrating immune compartment are modified upon mAb treatment; in particular, several studies reported that intratumoral and circulating NK cell numbers increase upon tumor-targeting mAb-based combination regimens and that this correlated with favorable outcome [95,96,97,98,99]. Moreover, the observation that anti-lymphoma activity of rituximab was improved in the presence of anti-KIR mAb (IPH2102-lirilumab), in a syngeneic murine lymphoma model [100], and the evidence that NKG2A blockade by specific mAb (IPH2201-monalizumab) potentiated cetuximab-dependent NK antitumor functions [101], together with the observation that Treg-mediated suppression of ADCC correlates with a lower clinical efficacy in cetuximab-treated HNSCC patients [102], further stress the concept that CD16-dependent NK cell effector functions heavily contribute to explain the therapeutic efficacy of tumor-targeting mAbs.

Despite widespread treatment successes, the development of resistance to therapeutic mAbs, leading to a substantial decrease in therapeutic efficacy and to a high relapse rate, is a major problem [103]. Besides tumor cell enhanced resistance to apoptosis and the presence of an immunosuppressive TME, several mechanisms may be responsible for the loss of ADCC efficacy. Antigen downmodulation, by internalization and/or Fc receptor-mediated trogocytosis, is a well-known mechanism of acquired resistance to rituximab [104,105,106,107]. Complement activation may represent an additional mechanism of resistance since depletion of the C3 component increased NK cell-mediated ADCC against rituximab-coated target cells and improved the efficacy of antibody therapy in an in vivo model [108]. Further, the persistent contact with mAb-opsonized cells induces CD16 downmodulation by either receptor internalization or disintegrin and metalloproteinase (ADAM)17-dependent shedding and represents a well-known mechanism, leading to a reduced ability to execute ADCC [109,110]. Moreover, our studies have provided evidence that, under conditions of chronic CD16 engagement, the activation of SHP-1 tyrosine phosphatase induces a wide-range functional exhaustion status, which hampers NK cell killing triggered by either CD16 or other activating receptors, such as NKG2D and NKp46 [111]; along this line, ex vivo analysis of rituximab-treated DLBCL patients revealed a marked and prolonged therapy-induced reduction of both “natural” and CD16-dependent NK cytotoxic activities, accompanied by the downmodulation of CD16 and NKG2D-activating receptors [112].

## 4. NK Cells as Players in Tumor-Targeting mAb-Dependent “Vaccinal Effect”

Passive immunotherapy with therapeutic mAbs can facilitate the development of active antitumor immunity by inducing both T-cell-mediated responses and endogenous humoral immunity [23]. Such long-lasting mAb-induced antigen-specific immune responses have been documented in several studies and defined as the “vaccinal effect”. The observation that the frequency of idiotype-specific T cells increases after rituximab treatment of FL patients provided the “proof of principle” for the ability of passive immunotherapy to elicit an active antigen-specific cellular response [113]. Several lines of evidence showed that trastuzumab therapy promotes or amplifies anti-HER2 antibody responses and also induces epitope spreading; notably, these studies also suggested that the mAb-dependent vaccinal effect is relevant in prolonging patient’s survival [114,115,116,117]. Although the mechanisms that explain the vaccinal effect have not been fully clarified, the enhanced FcγR-dependent uptake of tumor antigen/mAb immune complexes by DC is considered relevant [118,119,120,121,122]. NK cells, besides promoting the release of tumor antigens through ADCC, may broadly contribute to the mAb-dependent vaccinal effect. NK/DC crosstalk has been shown to crucially contribute to the development of antitumor adaptive responses. Indeed, intratumoral NK-derived cytokines and chemokines promote DC recruitment and survival at the tumor site [18,123,124]; moreover, NK cell-derived IFNγ efficiently promotes DC maturation (editing), which contributes to the development of a Th1-oriented response and amplifies the programming of CTL responses [18,40,125,126,127,128,129]. NK cell’s role in supporting DC-dependent T-cell responses has been demonstrated in cetuximab-treated HNC patients [130]; further, anti-CD20 mAb-induced Th1 response was shown to require the NK cell-dependent activation of DC in a mouse model [131]. Collectively, these results underlie the contribution of NK cells to the capability of tumor-targeting mAbs to tip the balance in favor of antitumor immune contexture, in which adaptive cellular immunity can develop.

## 5. Strategies Aimed at Potentiating NK Cell Response to Tumor-Targeting mAbs

### 5.1. Strategies That Improve mAb Interaction with CD16

The genetic manipulation and the glycoengineering of the Fc region of tumor-targeting mAbs have been exploited in order to modulate their interaction with activating and inhibitory members of the FcγR family. Selected amino acid substitutions, or the removal of fucose residues, increase IgG affinity for CD16 and result in enhanced NK cell-mediated ADCC and serial killing of multiple mAb-coated target cells [132,133,134,135,136,137,138]. Obinutuzumab, the first glycoengineered mAb approved for clinical use, is a humanized Fc-defucosylated anti-CD20 mAb with increased CD16 binding affinity [83,139,140,141]. Clinical trials in chronic lymphocytic leukemia (CLL) and FL patients showed superior efficacy of obinutuzumab vs. rituximab in combination with chemotherapy [142,143,144,145]. Compared to rituximab, obinutuzumab elicited stronger ADCC, which was insensitive to CD16-V158F polymorphism, showed more potent antitumor activity in lymphoma xenograft mouse models, as well as reduced sensitivity to the inhibitory effects of KIR/MHC-I ligation [134,146,147]. Interestingly, we documented that pre-exposure to obinutuzumab-coated targets primes NK cells to provide an enhanced IFNγ response to activating receptors or cytokine stimulation, and that this hyperresponsive condition is associated with microRNA-155 upregulation and to the amplification of PI3K/mTOR pathway [148,149].

**Figure 1:**
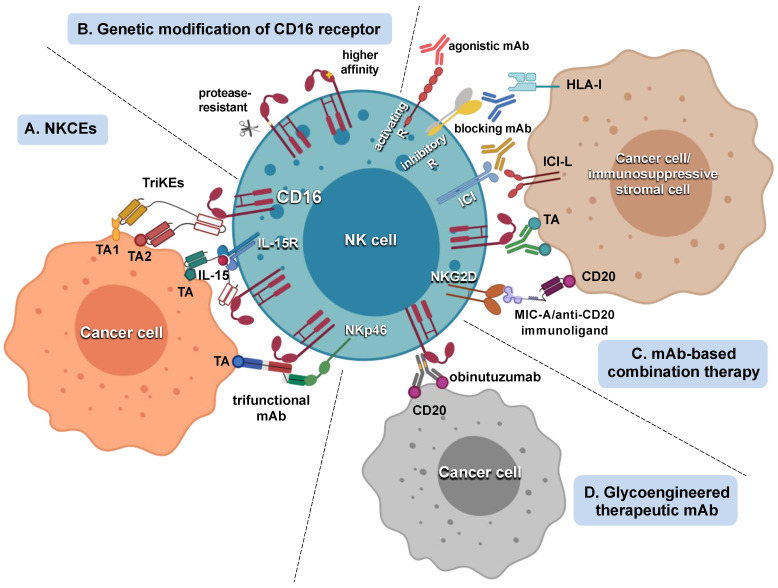
Overview of the main strategies aimed at potentiating therapeutic mAb-mediated CD16 activation of NK cells: (**A**) NK cell killer engagers (NKCEs). Recombinant chimeric molecules, such as trispecific killer engagers (TriKEs), which are composed of three single-chain variable fragments (scFv) recognizing CD16 and two different tumor-associated anti-gens (TA); two scFv fragments, linked to IL-15 moiety; trifunctional mAbs composed of two Fab fragments directed against a TA and NKp46, and a high-affinity Fc region, to target CD16. (**B**) Genetic modifications of CD16 gene to abolish sensitivity to ADAM-17-mediated proteolytic cleavage or to enhance affinity for IgG Fc region. (**C**) Tu-mor-targeting mAbs can be combined with agonistic mAbs targeting activating receptors; blocking mAbs targeting HLA-I-specific inhibitory receptors or immune checkpoints (ICI); immunoligands targeting a TA and engaging an ac-tivating receptor (MIC-A/anti-CD20). (**D**) Anti-CD20 humanized mAb obinutuzumab has been Fc glycoengineered to increase binding affinity for CD16 receptor. Figures were created with BioRender software (BioRender, Toronto, ON, Canada).

Another clinically available Ig-based strategy to efficiently redirect NK cell cytotoxicity toward tumor cells is represented by the generation of bispecific killer engagers (BiKEs) or trispecific killer engagers (TriKEs). Such small constructs (50–75 kDa) are often composed of a single-chain variable fragment (scFv) of an anti-CD16 Ab connected to the scFv of one (BiKE) or two (TriKE) tumor antigen-specific antibodies. These constructs bridge NK cells and tumor cells and allow high-affinity engagement of CD16, thus overcoming the limitations represented by receptor polymorphisms [4,16,17,26,80,150,151,152,153,154,155]. Further modifications of this strategy are represented by the possibility to target two different epitopes of the same tumor antigen, thus strengthening the avidity of NK-tumor interaction [156,157]. Although endowed with a shorter serum half-life [158], BiKEs and TriKEs display advantages with respect to standard therapeutic mAbs, in terms of better biodistribution, lower immunogenicity, and more rapid generation. Among others, CD16xCD133 bispecific killer-cell engager bears important therapeutic potential, given its ability to target the drug-resistant CD133^+^ cancer stem cell population [159]; another added value of these constructs is represented by their capability to redirect NK cell killing against immunosuppressive components in the TME, as in the case of the CD16xCD33 BiKE that targets MDSC [153]. Further modifications of this platform are represented by TriKEs where one tumor-antigen-specific moiety is substituted by, or implemented with (to generate a TetraKE), an activating cytokine; indeed, IL-15-containing KEs have proved to promote in vivo persistence, activation, and survival of NK cells, and also rendered NK cells more resistant to immunosuppressive factors in the TME [160,161,162,163,164,165]. Finally, the combination with inhibitors of ADAM-17, responsible for the CD16 shedding on activated NK cells [109,110,151], enhanced CD16xCD33 BiKE-dependent response against primary AML cells [166] (Table 1 and Figure 1).

### 5.2. Strategies That Amplify NK Cell Responsiveness to CD16 Engagement

Although CD16 engagement is capable of inducing NK cell activation and full display of effector functions by itself, the cooperation with activating receptors that recognize tumor ligands may increase the therapeutic efficacy of tumor-targeting mAbs, especially under conditions of suboptimal CD16 aggregation [6,30,168]. Several studies showed that complementary signals, provided by activating NK receptors upon ligand recognition or agonistic mAb binding, may potentiate NK cell effector functions against therapeutic mAb-opsonized tumor cells [169,170]. This body of evidence lends ground to the design of mAb combination strategies [168,171,172,173] and to the generation of bispecific molecules that associate a tumor-specific scFv with cognate ligands for either NKG2D or NKp30, and that synergistically augment ADCC [174]. Along this line, trifunctional NK-cell engagers (NKCEs), consisting of two mAb fragments targeting the NK activating receptor NKp46 and a specific tumor antigen, joined to an Fc fragment optimized for CD16 binding, were shown to promote tumor clearance and improve NK cell tumor infiltration; notably, these constructs resulted more efficient than intact tumor-targeting mAbs in vivo in mouse models [175]. Similarly, an anti-MIC-A/B mAb that blocks NKG2D ligand shedding from tumor cells potentiated NK cell-mediated tumor rejection in a CD16- and NKG2D-dependent manner in a preclinical model [176].

On the other hand, CD16-dependent NK cell activation and effector functions are under the control of MHC class I-specific and -nonspecific inhibitory receptors. Several reports have shown that the functional outcome of CD16-dependent tumor-targeting mAb activation may be negatively affected by the simultaneous engagement of MHC-I inhibitory receptors in vitro [147,177,178,179,180,181]. The lack of inhibitory KIR-ligand interaction was associated with improved clinical outcomes in neuroblastoma and lymphoma patients treated with anti-GD2 mAb or rituximab, respectively [180,181,182]. Following this evidence, an anti-KIR mAb (IPH2102-lirilumab) was shown to increase anti-CD20 or anti-CD38 mAb-triggered ADCC in vitro, and therapeutic mAb efficacy in a mouse model of B lymphoma [100,101,178]; following these preclinical results, lirilumab/rituximab combination has been preliminarily tested in CLL patients. Along the same line, NKG2A blockade by specific mAb (IPH2201-monalizumab), combined with anti-EGFR cetuximab [101], is currently under a phase II trial and has provided encouraging results for the treatment of HNSCC patients. A further combination strategy to enhance antitumor mAb-triggered NK cell functions relies on the blockade of immune checkpoint inhibitory receptors. PD-1 or TIGIT blockade was shown to augment trastuzumab and cetuximab therapeutic efficacy in preclinical models and progressed to clinical trials in patients with advanced malignancies [35,36,37,182,183,184,185]; combinations aimed at blocking other immune checkpoints expressed in NK cells are currently under study and may soon approach the clinic [186] (Table 1 and Figure 1).

Finally, the increasingly understood immunomodulating capability of standard and novel chemotherapeutic approaches can be also exploited to potentiate NK cell response to tumor-targeting mAbs [187]. Lenalidomide and pomalidomide immunomodulatory drugs (IMiDs) increased rituximab-dependent NK cell ADCC, through mechanisms that have not been completely defined yet [188,189]. The combination of lenalidomide and rituximab has demonstrated synergy in FL, marginal zone and mantle cell lymphomas, and in CLL [187,190,191,192]. Further investigation of lenalidomide as an immunomodulator in solid tumors is warranted. In this context, a phase I trial evaluating the combination of lenalidomide and cetuximab for the treatment of advanced CRC and HNSCC demonstrated a dose-dependent increase in ADCC [193].

Collectively, the targeting of NK cell costimulatory or inhibitory receptors, or the drug-mediated manipulation of intracellular signaling pathways, are emerging as promising strategies to enhance the therapeutic efficacy of tumor-targeting mAbs, in the treatment of a variety of cancers. Further, the association with strategies aimed at reverting the immunosuppressive TME would also empower the functional response of NK cells to tumor-targeting mAb [194].

### 5.3. Tumor-Targeting mAb/Adoptive NK Cell Therapy Combination

The combination of NK cell-based adoptive therapy in order to improve the clinical efficacy of tumor-targeting mAbs is an emerging area of intervention. Several sources of NK cells can be exploited, such as peripheral blood, or umbilical cord NK cells; moreover, the choice of donors that maximize HLA/KIR mismatch, or with a high number of genes for activating MHC-specific receptors, can be beneficial [194]. Several currently ongoing clinical trials are testing the combination of tumor-targeting mAbs with the infusion of autologous or allogeneic HLA-haploidentical NK cell preparations in a variety of hematological or solid tumors (Table 2). Recently, a completed phase II clinical trial in refractory non-Hodgkin’s lymphoma, where patients received haploidentical donor NK cells in combination with rituximab and IL-2, registered 4 objective responses out of 14 patients [195]; additionally, two pilot/phase I trials combining anti-GD2 and haploidentical NK cells were recently reported [196,197]. In some cases, in vitro exposure to activating cytokines before the adoptive transfer can increase the functional capability and/or survival of NK cell populations. Interestingly, an ongoing phase I trial on patients affected by solid malignancies is testing the combination of trastuzumab or cetuximab with allogeneic NK cells, whose in vitro stimulation with a specific GSK3 inhibitor potentiates functional capability and acquisition of CD57 maturation marker [198]. The adoptive transfer of NK cells with enhanced effector functions, including natural cytotoxicity, ADCC, and cytokine responses could lead to better clinical efficacy (Table 2).

The possibility to manipulate NK cells genetically is actively explored through different technological approaches, although the available clinical data are still limited [28,185,199,200,201,202]. Genetic modifications that would improve NK cell responsiveness to tumor-targeting mAb stimulation involve the manipulation of CD16 receptor to enhance its affinity for IgG Fc and/or its resistance to proteolytic shedding [203,204,205,206,207,208] (Figure 1) and are variably associated with genetic modifications aimed at improving NK cell in vivo persistence/survival, trafficking to the tumor site, or resisting inhibitory components of the TME [204,209,210,211]. Among the various sources employed for adoptive therapy, induced pluripotent stem cells (iPSC)-derived NK cells are particularly promising since they are able to mediate both natural and ADCC cytotoxic activities, and the potential for expansion and persistence in vivo [212]; in this context, iPSC-derived NK cells, transduced with anti-CD19 chimeric antigen receptor (CAR) and a noncleavable CD16 mutant form, are currently under trial in combination with anti-CD20 mAb in patients affected by B cell malignancies [213,214] (Table 2).

Based on our abovementioned data [111], the genetic manipulation of NK cells with the aim of targeting the mechanism(s) responsible for the hyporesponsiveness induced by chronic engagement of CD16 receptor could represent an additional strategy to improve the efficacy of tumor-targeting mAb/NK cell-based adoptive therapy combination. Finally, the possibility to modify the immunosuppressive microenvironment could also lead to the potentiation of therapeutic efficacy of NK cell-based adoptive strategies [194].

## 6. Memory NK Cells as Emergent Effectors in mAb-Based Antitumor Approaches

The recently appreciated heterogeneity of NK cells, along with their plasticity and capacity to adapt to environmental conditions, may be exploited by novel approaches to improve the efficacy of tumor-targeting mAb-based therapies. In humans, the stable expansion of a defined subset within mature CD56^dim^CD16^+^ circulating NK cells has been solidly documented in a large fraction of healthy, human cytomegalovirus (HCMV)-seropositive individuals [39,215,216]. This subset, named “memory” (or “adaptive”), is found at a highly variable frequency in different individuals and is distinct from conventional NK cells in regard to the expression of surface receptors, transcription factors, and key components of cellular signaling. Among their peculiar phenotypic characteristics, memory NK cells preferentially express CD94/NKG2C, the HLA-E-specific activating receptor, associated with CD57 terminal maturation marker, and display a skewed KIR profile [39,215,216]; additionally, memory NK cells are characterized by the absence of FcεRIγ signaling adaptor, variably associated with the lack of other intracellular signaling intermediates, such as SYK tyrosine kinase, EAT adaptor, and PLZF and IKF2 transcription factors [217,218,219,220,221,222]. This peculiar expression pattern mostly depends on a distinctive epigenetic profile that resembles that of memory CD8^+^ T cells [217,218,222]. Memory NK cell most relevant functional feature is represented by a markedly higher functional response to CD16 stimulation [217,218,219,220,221,222]; in particular, the capability to produce a high amount of IFNγ depends on the demethylation of the conserved noncoding sequence (CNS) 1 in the IFNG locus [223].

The close association with HCMV serostatus indicates that this NK cell subset represents a key component in the remodeling of the entire immune system that occurs in response to HCMV infection [39,224,225]. Clinical data that support a decisive role for CMV in driving the expansion of memory NK cells in vivo are provided by studies that reported a strong increase of NKG2C^+^ NK cells in solid organ transplanted patients, during acute CMV infection [226], or in bone marrow transplant recipients, following acute HCMV infection or reactivation [227,228,229,230]. The mechanisms regulating the persistence and amplitude of the memory NK cell pool in different individuals are still mostly unknown. The expansion of NKG2C^+^ NK cells has been documented in patients with other viral (hepatitis B virus, hepatitis C virus, human immunodeficiency virus, hantavirus [224,231,232,233,234]) or nonviral infections [235] and shown to occur almost exclusively in CMV-seropositive individuals.

Although the HCMV-dependent molecular signals that drive memory NK cell proliferation remain unknown, seminal data showed that CMV-infected cells can trigger the expansion of NKG2C^+^ NK cells in vitro [236,237,238], with the contribution of additional signals [239]. Interestingly, several reports have described the capability of CD16 stimulation by antiviral Ab-coated infected cells to drive the in vitro proliferation of memory NK cells [217,218].

The role exerted by memory NK cells in antitumor immunosurveillance still needs to be fully assessed; however, the expansion of NKG2C^+^CD57^+^ memory NK cells, which occurs after CMV reactivation, is associated with a significantly reduced relapse rate in bone marrow-transplanted leukemic patients [240]; this piece of evidence suggests that memory NK cells’ ability to recognize leukemic blasts may be effective in eradicating the minimal residual disease. Several still poorly characterized distinctive features of memory NK cells may predict a lower sensitivity to immunosuppressive signals in the TME; indeed, memory NK cells exhibited inherent resistance to Treg-mediated suppression, due to a lower expression of IL1R8 (IL-37R) and PD-1 inhibitory receptors [241]; on the same line, they were found to express lower levels of TIGIT immune checkpoint; as a consequence, memory NK cells were less sensitive to the inhibitory activity of CD155^+^ MDSC obtained from patients with myelodysplastic syndrome [242].

**Figure 2:**
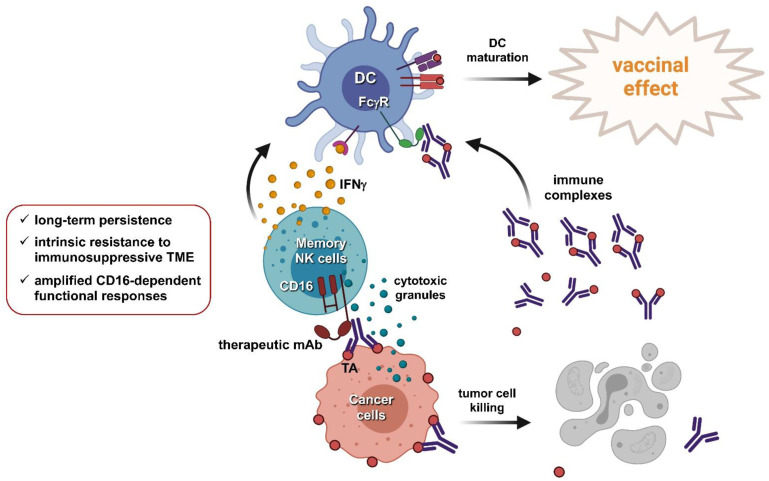
Memory NK cells as a tool to potentiate the clinical efficacy of tumor-targeting mAbs in combined therapeutic strategies. Endogenous or adoptively transferred memory NK cells may provide a heightened CD16-dependent response to tumor-targeting mAb, both in terms of ADCC-mediated tumor clearance and in the establishment of mAb-dependent tumor-specific adaptive responses (vaccinal effect). DC: dendritic cell. Figures were created with BioRender.

Several features render memory NK cells a potentially attractive contributor to the efficacy of mAb-based therapeutic strategies [243]. The capability of memory NK cells to provide an amplified functional response to CD16 cross-linking upon interaction with Ab-coated target cells is particularly relevant in this regard [217,218,219,220,221,222]. Our study revealed that upon stimulation with rituximab- or obinutuzumab-opsonized tumor cells, memory NK cells display a higher frequency of multifunctional IFNγ^+^CD107a^+^ cells with respect to the conventional population [244]. This enhanced responsiveness may impact the clinical efficacy of tumor-targeting mAb therapies, particularly in the context of the aforementioned vaccinal effect (Figure 2).

The first confirmation of the beneficial role of memory NK cells in sustaining therapeutic efficacy of tumor-targeting mAbs is provided by a recent report in which adaptive NK cells were shown to be important mediators of ADCC in daratumumab-treated multiple myeloma patients [245]. Along this line, memory NK cells were suggested to be refractory to the development of functional hyporesponsiveness after chronic CD16 engagement, when compared to what happens to conventional NK cells upon prior exposure to rituximab [111,246].

The capability of memory NK cells to proliferate in response to mAb-coated tumors may be instrumental in the design of strategies based on the adoptive transfer of memory NK cells combined with tumor-targeting therapeutic mAb. Indeed, the search for the stimuli that would allow memory NK cell in vitro expansion has been very intense in the last few years. Our own work demonstrated the capability of therapeutic mAb-opsonized targets to drive efficiently the selective in vitro expansion of memory NK cells from HCMV^+^ donors; in our conditions, the role of CD16 binding affinity for IgG Fc portion could also modulate the amplitude of memory NK cell proliferation [14,244]. Further, the already mentioned long-term persistence in vivo of memory NK cell pool [227,228,229,230] hints at a longer half-life of this NK cell subset; indeed, the analysis of patients with either paroxysmal nocturnal hemoglobinuria or GATA2 deficiency provided evidence of much more marked longevity of memory NK cells, when compared with their conventional counterpart [247,248]. The higher expression levels of the antiapoptotic molecule Bcl-2 ([221] and C. Capuano et al., unpublished results), along with a distinct metabolic profile [249], provide the molecular basis for this longevity. Additionally, therapeutic strategies that combine the adoptive transfer of allogeneic memory NK cell with tumor-targeting mAb administration could take advantage of the skewed repertoire of KIR expressed by memory NK cells, with the aim to select donor/recipient combinations that would lower the interference of MHC-I-specific inhibitory receptors on CD16-dependent activation [250]. Finally, a very recent report demonstrates that memory NK cells retain their distinctive functional characteristics after genetic manipulation [251], while a seminal study opens to the possibility to generate memory NK cells in vitro through genetic manipulation of conventional NK cells [252]. Another favorable characteristic of memory NK cells is represented by the fact that the enhanced capability to produce cytokines, particularly IFNγ, is under the control of CD16-initiated signals, thus strictly dependent on the availability of the tumor-targeting mAb. The possibility to modulate mAb dosing and administration schedule would thus safeguard against the risk of possibly excessive and harmful production of cytokines, as observed in the case of other adoptively transferred antitumor cell types, such as CAR-T cells. On the other hand, memory NK cells display a lower sensitivity to IL-12 and IL-18, due to lower receptor expression levels. It is anticipated that this peculiarity would render memory NK cells less susceptible to dysregulation in a strongly pro-inflammatory microenvironment.

## 7. Conclusions

Tumor-targeting mAbs represent an “arsenal” in the treatment of several hematologic and solid tumors and the first immunotherapeutic approach approved for clinical practice. NK cell effector functions are powerfully unleashed through CD16 engagement by IgG-coated tumor cells; thus, much interest fuels novel developments aimed at potentiating this tool in order to improve tumor-targeting mAb therapeutic efficacy. This can be accomplished either by improving CD16 interaction with mAbs or by combining tumor-targeting mAbs with agents capable of acting on stimulatory receptors and/or interfering with inhibitory signals that characterize the immunosuppressive TME or by optimizing NK cell survival/proliferation, recruitment, and persistence in tumor sites. All these approaches can be applied in vivo and/or exploited by in vitro genetic manipulation of NK cells.

Here, we propose that memory NK cells may have considerable potential due to their distinctive characteristics, including long-term persistence and intrinsic resistance to the tumor-dependent immunosuppressive microenvironment. These features, along with their amplified responses to antibody-coated target cells and in vitro expansion capability via CD16, may well support tumor-targeting mAb-dependent tumor clearance and the generation of antitumor adaptive immune responses that may provide long-term protection of mAb-treated patients. Further studies are needed to optimize the clinical application of memory NK cells. A better definition of their heterogeneity and role in mAb therapeutic efficacy, the possibility for their in vitro manipulation, or selective expansion under GMP-compliant conditions will be instrumental for better exploitation of NK cell memory for the ultimate benefit of treating cancer patients.

## Figures and Tables

**Table 1:** Selected ongoing clinical trials aimed at potentiating endogenous NK cell response to tumor-targeting mAbs.

Agent	Target	Malignancy	Study	ClinicalTrials.gov Identifier: Trial Number
**NK cell engager**				
AFM13	CD16A, CD30	HL	Phase 2	NCT02321592
		CTCL	Phase 1/2	NCT03192202
		HL	Phase 1	NCT01221571
		PTCL; Transformed Mycosis Fungoides	Phase 2	NCT04101331
		HL, in combination with anti-PD-1 antibody	Phase 1	NCT02665650
		R/R ALCL, R/R B-Cell NHL, R/R Classic HL, R/R Mycosis Fungoides, R/R PTCL	Phase 1	NCT04074746
AFM24	CD16A, EGFR	Advanced solid tumors	Phase 1/2	NCT04259450
GTB-3550	CD16, IL-15R, CD33	High-risk MDS; AML; Systemic Mastocytosis; Mast Cell Leukemia	Phase 1/2	NCT03214666
DF1001	CD16, HER2	Advanced stage HER2^+^ solid tumors, as monotherapy or in combination with anti-PD-1 mAb	Phase 1/2	NCT04143711
**Antibody combination**				
Rituximab plus Lirilumab	CD20, KIR	R/R or high-risk untreated patients with CLL	Phase 2	NCT02481297
Trastuzumab plus Monalizumab	HER2, NKG2A	Breast cancer	Phase 2	NCT04307329
Cetuximab plus Monalizumab	EGFR, NKG2A	Head and neck neoplasms	Phase 1/2	NCT02643550
Cetuximab plus Monalizumab	EGFR, NKG2A	HNSCC	Phase 3	NCT04590963
Cetuximab plus Pembrolizumab	EGFR, PD-1	HNSCC, Lip SCC, Oral Cavity Cancer	Phase 2	NCT03082534
Cetuximab plus Nivolumab	EGFR, PD-1	R/R or stage IV Head and Neck Squamous Cell Carcinoma, in combination with Paclitaxel	Phase 2	NCT04282109
Cetuximab plus Avelumab	EGFR, PD-L1	Squamous Cell Anal Carcinoma	Phase 2	NCT03944252

ALCL, anaplastic large cell lymphoma; AML, acute myelogenous leukemia; CLL, chronic lymphocytic leukemia; CTCL, cutaneous T-cell lymphoma; HL, Hodgkin’s lymphoma; HNSCC, head and neck squamous cell carcinoma; MDS, myelodysplastic syndromes; NHL, non-Hodgkin’s lymphoma; PTCL, peripheral T-cell lymphoma; R/R, relapsed and/or refractory; SCC, squamous cell carcinoma. Source: https://clinicaltrials.gov/ [167].

**Table 2:** Selected ongoing clinical trials employing adoptively transferred NK cell products to potentiate tumor-targeting mAb therapeutic efficacy.

Agent	Malignancy	Study	ClinicalTrials.gov Identifier: Trial Number
Autologous, in vitro-expanded NK cells	R/R neuroblastoma, in combination with dinutuximab anti-GD2 mAb and chemotherapy	Phase 1	NCT04211675
Autologous, in vitro-expanded NK cells (CELLPROTECT)	Post-transplant maintenance of MM, in combination with isatuximab anti-CD38 mAb	Phase 2	NCT04558931
Autologous, in vitro-expanded NK cells (SNK01)	Advanced/metastatic HER2- or EGFR-expressing cancers, in combination with trastuzumab or cetuximab anti-HER2 mAbs	Phase 1/2a	NCT04464967
autologous, in vitro-expanded NK cells	R/R neuroblastoma, in combination with Ch14.18 anti-GD2 mAb and lenalidomide	Phase 1	NCT02573896
Third-party in vitro-expanded (IL-21) NK cells	R/R CTL or ATL, in combination with mogamulizumab anti-CCR4 mAb	Phase 1	NCT04848064
HLA-mismatched NK cells	High-risk neuroblastoma, in combination with Hu3F8 anti-GD2 mAb and IL-2	Phase 1	NCT02650648
HLA-haploidentical in vitro-expanded NK cells	Neuroblastoma or R/R neuroblastoma, in combination with hu14.18-IL2 immunocytokine (anti-CD2 mAb linked to IL-2)	Phase 1	NCT03209869
iPSC-derived NK cells expressing high affinity, noncleavable CD16 (FT516)	R/R B cell lymphoma, in combination with rituximab or obinutuzumab anti-CD20 mAbs and IL-2	Phase 1/1b	NCT04023071
iPSC-derived NK cells expressing high affinity, noncleavable CD16 (FT516)	Recurrent gynecologic cancers, in combination with enoblituzumab anti-B7-H3 mAb and IL-2	Phase 1	NCT04630769
iPSC-derived NK cells expressing high affinity, no-cleavable CD16 and IL-15 receptor fusion (IL-15RF), CD38 *ko* (FT538)	R/R MM, in combination with daratumumab anti-CD38 or elotuzumab anti-SLAMF7 mAbs	Phase 1	NCT04614636
Anti-CD19 CAR-transduced iPSC-derived NK cells, expressing high affinity, noncleavable CD16 and IL-15 receptor fusion (IL-15RF) (FT596)	R/R CLL or B cell lymphoma, in monotherapy or in combination with rituximab or obinutuzumab anti-CD20 mAbs	Phase 1	NCT04245722
Allogeneic, in vitro-expanded and terminally differentiated NK cells (FATE-NK100)	Advanced-stage solid tumors, in monotherapy or in combination with cetuximab or trastuzumab anti-HER2 mAbs	Phase 1	NCT03319459

ATL, adult T-cell leukemia/lymphoma; CLL, chronic lymphocytic leukemia; CTCL, cutaneous T-cell Lymphoma; iPCS, induced pluripotent stem cell; ko, knockout; MM, multiple myeloma; R/R, relapsed and/or refractory. Source: https://clinicaltrials.gov/ [167].

## Abbreviations

ADCC	antibody-dependent cell-mediated cytotoxicity
BiKE	bispecific killer cell engager
TriKE	trispecific killer cell engager
CLL	chronic lymphocytic leukemia
CRC	colorectal cancer
CSC	cancer stem cells
DC	dendritic cell
(ADAM)17	disintegrin and metalloproteinase
FcγR	Fc gamma receptor
FL	follicular lymphoma
ITAM	immunoreceptor tyrosine-based activation motif
HCMV	human cytomegalovirus
HNSCC	head and neck squamous cell carcinoma
HSCT	hematopoietic stem-cell transplantation
IFNγ	interferon gamma
KIR	killer-cell immunoglobulin-like receptor
mAb	monoclonal antibody
NCR	natural cytotoxicity receptor
NK	natural killer
NKCE	NK cell engagers
NKG2D	natural killer group 2D
TA	tumor-associated antigen
TME	tumor microenvironment
